# Prescription of Chinese Herbal Medicine and Selection of Acupoints in Pattern-Based Traditional Chinese Medicine Treatment for Insomnia: A Systematic Review

**DOI:** 10.1155/2012/902578

**Published:** 2012-11-28

**Authors:** Wing-Fai Yeung, Ka-Fai Chung, Maggie Man-Ki Poon, Fiona Yan-Yee Ho, Shi-Ping Zhang, Zhang-Jin Zhang, Eric Tat-Chi Ziea, Vivian Wong Taam

**Affiliations:** ^1^Department of Psychiatry, University of Hong Kong, Pokfulam Road, Hong Kong; ^2^School of Chinese Medicine, Hong Kong Baptist University, Hong Kong; ^3^School of Chinese Medicine, University of Hong Kong, Hong Kong; ^4^Chinese Medicine Section, Hospital Authority, Hong Kong

## Abstract

Traditional Chinese medicine (TCM) treatments are often prescribed based on individuals' pattern diagnoses. A systematic review of Chinese and English literatures on TCM pattern differentiation, treatment principle, and pattern-based treatment for insomnia has therefore been conducted. A total of 227 studies, 17916 subjects, and 87 TCM patterns were analyzed. There was a limited consistency in pattern-based TCM treatment of insomnia across practitioners. Except for Gui Pi Tang, An Shen Ding Zhi Wan, and Wen Dan Tang which were used more commonly for *deficiency of both the heart and spleen*, *internal disturbance of phlegm-heat*, and *qi deficiency of the heart and gallbladder*, respectively, the selection of herbal formula for other patterns and pattern-based prescription of individual herbs and acupoints were not consistent. Suanzaoren (Semen Z. spinosae), Fuling (Poria), Yejiaoteng (Caulis P. multiflori), Gancao (Radix Glycyrrhizae), Baishao (Radix P. alba), Shenmen (HT7), Yintang (EX-HN3), Sanyinjiao (SP6), Baihui (GV20), Anmian (EX-HN22), and Sishencong (EX-HN1) were commonly used, but nonspecifically for many patterns. Treatment principles underlying herb and acupoint selection were seldom reported. Although many studies were reviewed, the study quality and diagnostic process were inadequate. More high quality studies are needed to examine the additional benefits of pattern differentiation and pattern-based TCM treatment.

## 1. Introduction

Insomnia is a frequent clinical complaint. Nearly one-third of the general population worldwide presents with insomnia symptoms, and the prevalence of insomnia symptoms accompanied by daytime consequences is approximately 9–15% [1]. Insomnia is a risk factor of a number of negative health consequences, including major depression, suicide, anxiety disorders, and alcohol and substance abuse or dependence [2]. Effective pharmacological treatments for insomnia are available. However, their uses are limited due to concerns about long-term efficacy and potential for dependence, abuse, and adverse effects [3]. Although psychological and behavioral therapies have empirical evidence for insomnia [4], they have remained underused because of the time-intensive nature and treatment nonadherence. Since the currently available treatments have their limitation, complementary and alternative medicine has been sought to treat insomnia. According to a national survey, over 1.6 million adults in the United States used complementary and alternative medicine to treat insomnia in the year 2002 [5].

Traditional Chinese medicine (TCM), a form of complementary and alternative medicine, is one of the oldest medical systems in the world. The use of TCM for insomnia can be traced more than 2000 years ago in Chinese medical texts [6, 7]. In clinical practice, TCM treatments are individualized according to the TCM pattern diagnosis. TCM pattern, in terms of TCM theory, is defined as a diagnostic conclusion of the pathological changes of a disease state based on an individual's symptoms and signs, including pulse form and tongue appearance [8]. The patterns of bodily disharmony can be described in terms of eight major parameters: *yin* and *yang*, *external* and *internal*, *hot* and *cold*, and *excess* and *deficiency*. Additional systems, such as *qi*, *blood* and *body-fluid* differentiation, and *zang fu* (organ) differentiation are also used [9]. For a given disease there may be different presentations of various symptoms and signs; hence, many TCM pattern diagnoses are possible and this forms the rationale that different TCM treatments can be given for the same disease. On the other hand, different diseases may be treated with the same TCM treatment if they share the same pattern. The TCM patterns used today and the selection of herbal formulas and acupoints for specific TCM patterns are developed based on clinical experience, ancient medical texts, and some recently published TCM textbooks. It has to be acknowledged that most TCM concepts and therapies have not yet been proven by scientific method.

Although it is believed that pattern-based TCM treatment will provide better efficacy, previous studies regarding the additional benefit of TCM pattern differentiation are scarce. One randomized controlled trial (RCT) found that the therapeutic effect of Chinese herbal treatment according to TCM pattern was more sustainable than the standard formula in treating irritable bowel syndrome [10]. It has also been reported in rheumatoid arthritic patients that TCM pattern diagnoses can guide the use of Western medicine [11]. However, other RCTs found that the efficacy of pattern-based individualized acupuncture was not superior to standardized acupuncture for chronic shoulder pain [12], chronic back pain [13], and hypertension [14].

A previous survey in a Chinese medicine clinic in Taiwan has summarized the commonly prescribed single Chinese herbs and herbal formulas for insomnia [15]. Another study has reviewed the randomized controlled trials of acupuncture for insomnia and determined the most frequently used acupoints [16]. To the best of our knowledge, there has been no systematic review on the TCM patterns for insomnia. Information regarding pattern-based TCM treatments for insomnia is also lacking. Given the high prevalence of insomnia in the general population and its frequent presentation to TCM practitioners, it is important to review the current application of syndrome differentiation in TCM treatments for insomnia. The objectives of this paper were (1) to summarize the commonly diagnosed TCM patterns in patients with insomnia and (2) to find out the current practice of pattern-based TCM treatments for insomnia.

## 2. Method

The present study was part of our systematic review on TCM treatments for insomnia. Chinese herbal medicine, traditional needle acupuncture, and variants of acupuncture, including acupressure, moxibustion, transcutaneous electrical nerve stimulation, auricular acupressure, and reflexology were reviewed [17]. We searched the MEDLINE, EMBASE, Cochrane Central Register of Controlled Trials, Cumulative Index to Nursing and Allied Health Literature, and Allied and Complementary Medicine from inception to May 2010 using the grouped terms (insomnia* or wakeful* or sleepless* or sleep*) and ((acupuncture* or acupoints* or acupressure* or meridian* or transcutaneous electrical nerve stimulation or TENS or moxibustion or tuina) or (herb* or herbal medicine* or traditional Chinese medicine* or TCM)). The search also included China Academic Journals Full-Text Database, China Proceedings of Conference Full-Text Database, Chinese Biomedical Literature Database, China Doctor Dissertations Full-Text Database, China Master Theses Full-Text Database, Chinese Science and Technology Documents Database, Chinese Dissertation Document Bibliography Database, Chinese Academic Papers Conference Database, Digital Periodical, and Taiwan Electronic Periodical Services. The reference lists of the retrieved papers were further searched for relevant articles.

### 2.1. Selection Criteria

Studies included in this paper were case series or clinical trials that described TCM patterns of subjects who received TCM treatment for their chief complaint of insomnia. In order to obtain a full coverage of the topic, we had not set any specification for sampling procedure, treatment method, outcome measure, and study quality. In addition, to derive a general picture of TCM pattern utilization, studies were excluded if they (1) had less than 30 subjects; (2) examined male or female only; (3) focused on subjects aged below 18 or above 70 years; (4) focused on a specific medical and psychiatric condition, a particular life transition period, or a specific TCM pattern; (5) had no statistical information regarding the frequency of individual TCM pattern; or (6) were duplicated publications. A sample size of 30 is considered as the minimum to examine the TCM pattern diagnosis in individuals with insomnia.

### 2.2. Data Extraction Process

Two authors (W. Yeung and M. Poon) searched the databases and selected the relevant publications independently. If there were any disagreements about the eligibility of a study, the two authors would check the study against the inclusion and exclusion criteria, discuss its eligibility, and come to a further decision. One author (M. Poon) extracted the data and the other (W. Yeung) checked the extracted data. For each study, the following variables were extracted: study design, sample size, mode of recruitment, sampling and diagnostic procedure, inclusion and exclusion criteria, and participants' characteristics including age, gender, and duration of insomnia. TCM patterns, treatment principles, treatment regimen, and outcome of TCM treatments were obtained. In a majority of the reviewed studies, effective rate was the only outcome measure. The definition of effective rate was not standardized, but it was most often defined as the proportion of subjects who had at least some improvement of sleep or at least 30% reduction in the severity of insomnia by rating scales. We calculated the mean, standard deviation, and 95% confidence interval of dichotomous outcome measure. All Chinese to English translations were deduced primarily from the *World Health Organization* (*WHO) International Standard Terminologies on Traditional Medicine in the Western Pacific Region* [8], and additionally from *Traditional Chinese Internal Medicine* [18], a commonly used English-language TCM textbook in China. 

### 2.3. Quality Assessment

For RCTs of Chinese herbal medicine, methodological quality will be assessed using Jadad scale. The Jadad scale evaluates a study in terms of the description of randomization, blinding, and dropouts. Points are awarded if the study is described as randomized, 1 point; has appropriate randomization method, 1 point; is described as double-blind, 1 point; uses appropriate blinding method, 1 point; has description of withdrawals and dropouts, 1 point. For RCTs involving acupuncture, a modified Jadad scale will be used. The modified Jadad scale takes into consideration the difficulty in blinding the acupuncturist to the treatment process. Points are awarded if the study is described as randomized, 1 point; has appropriate randomization method, 1 point; subjects blinded to intervention, 1 point; evaluator blinded to intervention, 1 point; has description of withdrawals and dropouts, 1 point [19, 20]. Both the Jadad scale and modified Jadad scale scores range from 1 to 5; RCTs with a score from 3 to 5 are regarded as better quality trials.

### 2.4. Statistical Analysis

We used SPSS version 16.0 for statistical analysis. Effective rate was summarized using mean (SD) and 95% confidence intervals (CIs). 

## 3. Results

**Figure 1:**
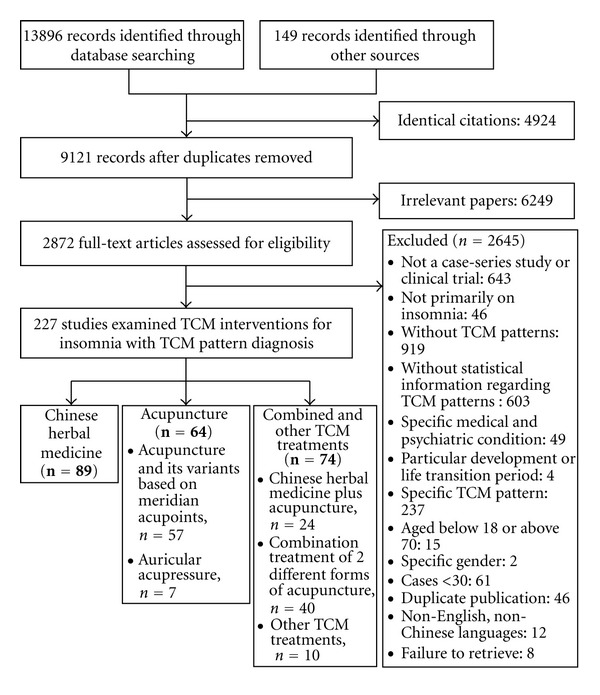
Study selection flowchart.

The search yielded 14045 potential titles for review, of which 4924 were duplicate records and 6249 were excluded for reasons of irrelevance. The full text of 2872 articles was retrieved for detailed assessment, of which 2645 were excluded for various reasons (Figure 1). Of the 227 studies which fulfilled the inclusion and exclusion criteria, 89 were on Chinese herbal medicine, 64 on acupuncture, 40 on a combination of two acupuncture treatments, 24 on a combination of Chinese herbal medicine and acupuncture, and 10 on other TCM treatments. Sample size of the 227 studies ranged from 30 to 400. TCM diagnosis was available in 17916 subjects. A total of 87 different TCM diagnoses were examined in the 227 studies. The most common pattern was *deficiency of both the heart and spleen*, which was diagnosed in 4056 subjects or 22.6% of the 17916 subjects; it was followed by *hyperactivity of fire due to yin deficiency* (*N* = 2926, 16.3%), *liver-qi stagnation transforming into fire* (*N* = 1817, 10.1%),* heart-kidney noninteraction* (*N* = 1157, 6.5%)*, internal disturbance of phlegm heat* (*N* = 904, 5.0%), *qi deficiency of the heart and gallbladder* (*N* = 838, 4.7%)*, liver fire flaming upward* (*N* = 456, 2.5%)*, heart deficiency with timidity* (*N* = 356, 2.0%), *disharmony between spleen and stomach *(*N* = 325, 1.8%), and *stomach disharmony* (*N* = 162, 0.9%). The top 10 TCM patterns accounted for 72.5% of the 17916 subjects (Table 1).

The criteria used for diagnosis of TCM pattern varied between studies. Twenty-one of the 227 studies (9.3%) were based on TCM textbooks, 17 (7.5%) studies used the Clinical Research Guidelines of New Chinese Herbal Medicine [21], 16 (7.0%) used the Criteria of Diagnosis and Therapeutic Effect of Diseases and Syndromes in TCM [22], 6 (2.6%) used some self-defined criteria, 3 (1.3%) adopted the criteria used by other TCM experts, and 1 used a combination of the Criteria of Diagnosis and Therapeutic Effect of Diseases and Syndromes in TCM and a textbook. One hundred and sixty-three studies (71.8%) did not report the diagnostic criteria used.

### 3.1. TCM Pattern and Chinese Herbal Medicine

Eighty-nine of the 227 included studies examined Chinese herbal medicine for insomnia: 54 of the 89 studies (60.7%) were case series, 8 (9.0%) were controlled studies, and 27 (30.3%) were RCTs. Of the 27 RCTs, 15 (55.6%) compared Chinese herbal medicine with Western medication, 9 (33.3%) with another Chinese herbal medicine, and 3 (11.1%) with Western medication and another Chinese herbal medicine. Twenty-six (96.3%) of the 27 RCTs were described as randomized but the randomization method, blinding, and dropouts were not presented; hence, these 26 RCTs had a Jadad score of 1. Only 1 (3.7%) of the 27 RCTs had appropriate randomization and double-blind design and obtained a Jadad score of 3. 

Gui Pi Tang or its modification was the most frequently used Chinese herbal formula for the treatment of insomnia in patients diagnosed with *deficiency of both the heart and spleen* (Table 2); for *hyperactivity of fire due to yin deficiency, *Huang Lian E Jiao Tang was the most frequently used formula. Modified Long Dan Xie Gan Tang and Dan Zhi Xiao Yao San were the most often used herbal formula for *liver-qi stagnation transforming into fire*. For *internal disturbance of phlegm heat*, the most frequently used formula was modified to Wen Dan Tang; lastly, for *qi deficiency of the heart and gallbladder*, modified An Shen Ding Zhi Wan was the most frequently used. There was a great variation in Chinese herbal treatment among patients diagnosed with *heart-kidney noninteraction* and *heart deficiency with timidity*, with no single formula being studied twice. Studies on *liver fire flaming upward*, *disharmony between spleen and stomach*, and *stomach disharmony* were so limited that reliable information was not possible (Table 2).

The TCM treatment principles and ingredients of the Chinese herbal formulas for treating insomnia are presented in Table 2. Due to a wide variation in the composition of herbal formulas, we only listed those individual herbs that were used in at least 20% of the studies on the TCM pattern. Suanzaoren (Semen Z. spinosae), Danggui (Radix A. sinensis), Yuanzhi (Radix Polygalae), Dangshen (Radix Condonopsis), Huangqi (Radix Astragali), Fuling (Poria), Baizhu (Rhizoma A. macrocephalae), Yejiaoteng (Caulis P. multiflori), Fushen (Poria cum Radix Pini), Muxiang (Radix Aucklandiae), Longyanrou (Arillus longan), Honey-toasted Gancao (Radix Glycyrrhizae), Gancao (Radix Glycyrrhizae), Baishao (Radix P. alba), Dazao (Fructus Jujibae), Wuweizi (Fructus S. Chinensis), Hehuanpi (Cortex Albiziae), Baiziren (Semen Platycladi), and Shengjiang (Rhizoma Zingiberis) were common individual herbs for the treatment of insomnia in patients with *deficiency of both the heart and spleen, *and the most frequently used TCM treatment principle was to nourish* heart* and* spleen*. For* hyperactivity of fire due to yin deficiency*, Suanzaoren (Semen Z. spinosae), Shengdihuang (Radix Rehmanniae), Yejiaoteng (Caulis P. multiflori), Ejiao (Colla C. asini), Huanglian (Rhizoma Coptidis), Gancao (Radix Glycyrrhizae), Baishao (Radix P. alba), Hehuanpi (Cortex Albizziae), Fuling (Poria), Zhimu (Rhizoma Anemarrhenae), Wuweizi (Fructus S. Chinensis), Fushen (Poria cum Radix Pini), Yuanzhi (Radix Polygalae), and Huangbo (Cortex Phellodendri) were commonly used individual herbs for insomnia and the most frequently used TCM treatment principle was to nourish* yin* and suppress *fire*. For *liver-qi stagnation transforming into fire*, Chaihu (Radix Bupleuri), Zhizi (Fructus Gardeniae), Gancao (Radix Glycyrrhizae), Danggui (Radix A. sinensis), Suanzaoren (Semen Z. spinosae), Baishao (Radix P. alba), Yejiaoteng (Caulis P. multiflori), Longdancao (Radix Gentianae), Yujin (Radix Curcumae), Fuling (Poria), Shengdihuang (Radix Rehmanniae), Hehuanpi (Cortex Albizziae), Huangqin (Radix Scutellariae), Fushen (Poria cum Radix Pini), Zexie (Rhizoma Alismatis), Zhenzhumu (Concha Margaritifera), Danpi (Cortex Moutan), and Baizhu (Rhizoma A. macrocephalae) were more often used and the most frequently used TCM treatment principle was to soothe *liver* and purge *fire*. There was no single herb being used in more than 50% of the studies on *heart-kidney noninteraction, *indicating that the selection of Chinese herbs varied greatly among practitioners. Suanzaoren (Semen Z. spinosae), Fuling (Poria), Yejiaoteng (Caulis P. multiflori), Gancao (Radix Glycyrrhizae), and Baishao (Radix P. alba) were the most commonly used individual herbs, but they were also the most nonspecific and were used in most of the TCM patterns. 

The effective rate of pattern-based Chinese herbal medicine treatment for insomnia was provided in 38 of the 89 studies on Chinese herbal medicine (Table 3). Based on the findings from RCTs, the mean effective rates were similar across TCM patterns, with the highest mean effective rate of 95.0% for* liver-qi stagnation transforming into fire *and the lowest at 79.4% for *heart deficiency with timidity*. When the case series were examined, the mean effective rates in *liver-qi stagnation transforming into fire* and *heart deficiency with timidity *were very similar (79.1% and 77.8%, resp.).

### 3.2. TCM Pattern and Acupuncture-Related Treatments

Fifty-seven of the 227 reviewed studies examined acupuncture-related treatments, including traditional needle acupuncture (*n* = 35), acupressure (*n* = 12), moxibustion (*n* = 3), acupoint injection (*n* = 3), acupoint plaster (*n* = 3), and acupoint embedment (*n* = 1). Forty-one (71.9%) of the 57 studies were case series, 4 (7.0%) were controlled studies, and 12 (21.1%) were RCTs. Of the 12 RCTs, 6 (50.0%) compared acupuncture with Western medication, 3 (25.0%) with another acupuncture treatment, 1 (8.3%) with Chinese herbal medicine, 1 (8.3%) with sleep hygiene education, and 1 (8.3%) with sham acupuncture and another acupuncture treatment. Ten (83.3%) of the 12 RCTs were described as randomized but the randomization method, blinding, and dropouts were not presented; hence, these 10 studies had a modified Jadad score of 1. One RCT (8.3%) was described as randomized and included the dropouts; hence, a score of 2 was given. Another RCT (8.3%) described the randomization method, blinding, and dropouts but not evaluator blind; hence, a score of 4 was given.

Table 4 presents the acupoints that were commonly used for treating insomnia. There were only 7 studies on auricular acupressure; hence, the use of auricular points in TCM pattern-based treatment of insomnia was not presented. The treatment principles were also not presented because the number of studies with the data was too few, ranging from 0 to 3 in each TCM pattern. The commonly used acupoints in patients with *deficiency of both the heart and spleen* were Xinshu (BL15), Shenmen (HT7), Sanyinjiao (SP6), Pishu (BL20), Zusanli (ST36), Neiguan (PC6), Baihui (GV20), Yintang (EX-HN3), Anmian (EX-HN22), Sishencong (EX-HN1), Fengchi (GB20), and Zhongwan (CV12). For *hyperactivity of fire due to yin deficiency*, Shenmen (HT7), Sanyinjiao (SP6), Shenshu (BL23), Taixi (Kl13), Xinshu (BL15), Baihui (GV20), Neiguan (PC6), Zhongwan (CV12), Sishencong (EX-HN1), Qihai (CV6), Yintang (EX-HN3), Anmian (EX-HN22), Fengchi (GB20), Zhaohai (Kl6), and Guanyuan (CV4) were commonly used acupoints. Sanyinjiao (SP6), Taichong (LR3), Shenmen (HT7), Ganshu (BL18), Zhongwan (CV12), Neiguan (PC6), Qihai (CV6), Fengchi (GB20), Guanyuan (CV4), Baihui (GV20), Xiawan (CV10), Shenshu (BL23), and Yintang (EX-HN3) were commonly used acupoints in patients with* liver-qi stagnation transforming into fire*. The selection of acupoints in other TCM patterns is shown in Table 4. There was no acupoint being used in more than 50% of the studies on *liver-qi stagnation transforming into fire *and *heart-kidney noninteraction, *indicating that the selection of acupoints for these 2 TCM patterns varied greatly. Shenmen (HT7), Yintang (EX-HN3), Sanyinjiao (SP6), Baihui (GV20), Anmian (EX-HN22), and Sishencong (EX-HN1) were the most common acupoints for the treatment of insomnia.

The effective rate of pattern-based acupuncture-related treatments for insomnia was provided in 24 of the 64 studies on acupuncture-related treatments (Table 3). As there were few RCTs available for comparison, based on the case series findings, the mean effective rates of acupuncture for the different TCM patterns were similar, with the highest mean effective rate of 98.1% for *disharmony between spleen and stomach *and the lowest at 75.0% for *qi deficiency of the heart and gallbladder*.

### 3.3. TCM Pattern and Combined TCM Treatments 

#### 3.3.1. Chinese Herbal Medicine Plus Acupuncture-Related Therapies

Twenty-four studies examined Chinese herbal medicine plus acupuncture-related treatments for insomnia. Thirteen (54.2%) of the 24 studies were case series, 1 (4.2%) was a controlled study, and 10 (41.7%) were RCTs. Of the 10 RCTs, 5 (50.0%) used Chinese herbal medicine for comparison, 3 (30.0%) used Western medication, and 2 (20.0%) compared with usual care. All 10 RCTs had a modified Jadad score of 1 as they were only described as randomized, but the randomization method, blinding, and dropouts were not presented.

We only presented the results of Chinese herbal medicine plus traditional needle acupuncture in *deficiency of both the heart and spleen, hyperactivity of fire due to yin deficiency*, and *liver-qi stagnation transforming into fire*, because studies on other TCM patterns and other forms of acupuncture were too few to give reliable information (Table 5). For *deficiency of both the heart and spleen*, the commonly used herbal ingredients for insomnia were Suanzaoren (Semen Z. spinosae), Baizhu (Rhizoma A. macrocephalae), Danggui (Radix A. sinensis), Huangqi (Radix Astragali), Yuanzhi (Radix Polygalae), Muxiang (Radix Aucklandiae), Fuling (Poria), Longyanrou (Arillus Longan), Hehuanhua (Flos Albiziae), Dazao (Fructus Jujibae), Gancao (Radix Glycyrrhizae), Yejiaoteng (Caulis P. multiflori), Wuweizi (Fructus S. Chinensis), Renshen (Radix Ginseng), Fushen (Poria cum Radix Pini), Honey-toasted Gancao (Radix Glycyrrhizae), Chuanxiong (Rhizoma Chuanxiong), Ginger (Rhizoma Z. recens), Baiziren (Semen Platycladi), Dangshen (Radix Codonopsis), Chaihu (Radix Bupleuri) in combination with acupuncture at Shenmen (HT7), Sanyinjiao (SP6), Xinshu (BL15), Pishu (BL20), Neiguan (PC6), Zusanli (ST36), Sishencong (EX-HN1), and Baihui (GV20). The selection of herbs and acupoints combination and TCM treatment principles in other TCM patterns is shown in Table 5. Danggui (Radix A. sinensis), Chaihu (Radix Bupleuri), and Gancao (Radix Glycyrrhizae) and Shenmen (HT7), Sanyinjiao (SP6), Neiguan (PC6), Zusanli (ST36), Sishencong (EX-HN1), and Baihui (GV20) were the common Chinese herbs and acupoints, respectively, in the combined Chinese herbal medicine and traditional needle acupuncture treatment for insomnia in *deficiency of both the heart and spleen, hyperactivity of fire due to yin deficiency, *and* liver-qi stagnation transforming into fire*.

#### 3.3.2. Combination of Acupuncture Treatments

Forty studies examined the combination of 2 different acupuncture modalities for the treatment of insomnia. The TCM treatment principles were not presented due to the small number of studies with relevant information. Twenty-nine (72.5%) of the 40 studies were case series, 1 (2.5%) was a controlled study, and 10 (25.0%) were RCTs. Of the 10 RCTs, 5 (50.0%) used another acupuncture treatment for comparison, 4 (40.0%) used Western medication, and 1 (10.0%) used Chinese herbal medicine. Nine (90.0%) of the RCTs had a modified Jadad score of 1, and the other RCT (10.0%) presented the appropriate randomization method; hence, a score of 2 was given.

Only the studies that examined traditional needle acupuncture plus auricular acupressure are presented, because studies on other combinations are too few to give reliable information (Table 6). For *deficiency of both the heart and spleen*, the commonly used acupoints were Baihui (GV20), Sanyinjiao (SP6), Shenmen (HT7), Sishencong (EX-HN1), Neiguan (PC6), Xinshu (BL15), Zusanli (ST36), Pishu (BL20), Anmian (EX-HN22), Fengchi (GB20); while the commonly used auricular points were shenmen, subcortex, heart, spleen, sympathesis, kidney, brain, endocrine, neurasthenia point, stomach, liver, and occiput. The selection of acupoints and auricular points for other TCM patterns is shown in Table 6. Sanyinjiao (SP6), Baihui (GV20), Shenmen (HT7), Sishencong (EX-HN1), and Neiguan (PC6) and shenmen, subcortex, heart, kidney, sympathesis, spleen, and liver were the commonly used acupoints and auricular points, respectively, in the combined traditional needle acupuncture and auricular acupressure for insomnia.

## 4. Discussion

This study is the first systematic review of English and Chinese literature, involving 227 studies and 17916 subjects on the classification and treatment of insomnia using the TCM diagnostic system. We found that the TCM pattern diagnoses for insomnia were diverse, with a total of 87 different TCM patterns identified. *Deficiency of both the heart and spleen *was the most commonly used TCM pattern in subjects with insomnia, followed by *hyperactivity of fire due to yin deficiency*, *liver-qi stagnation transforming into fire*,* heart-kidney noninteraction, internal disturbance of phlegm-heat*,* qi deficiency of the heart and gallbladder*,* liver fire flaming upward, stomach disharmony*,* heart deficiency with timidity*, and *disharmony between spleen and stomach*. Pattern-based TCM treatment was more common in *deficiency of both the heart and spleen*, *internal disturbance of phlegm heat*, and *Qi deficiency of the heart and gallbladder*, but limited to the use of Chinese herbal medicine formula. Gui Pi Tang was the most frequently used herbal formula in *Deficiency of both the heart and spleen*; for *Internal disturbance of phlegm heat* and *qi deficiency of the heart and gallbladder*, it was Wen Dan Tang and An Shen Ding Zhi Wan, respectively. For other TCM patterns, no single formula could be regarded as commonly used across TCM practitioners. 

We could not identify certain combinations of herbal agents or acupoints being used for particular TCM patterns. Suanzaoren (Semen Z. spinosae), Fuling (Poria), Yejiaoteng (Caulis P. multiflori), Gancao (Radix Glycyrrhizae), and Baishao (Radix P. alba) and Shenmen (HT7), Yintang (EX-HN3), Sanyinjiao (SP6), Baihui (GV20), Anmian (EX-HN22), and Sishencong (EX-HN1) were the commonly used Chinese herbs and acupoints, respectively, and they were being used nonspecifically for treating insomnia in many different TCM patterns. Treatment principles underlying the selection of herbal combinations were seldomy reported and it was more obvious with pattern-based acupuncture studies. One explanation for the variability in pattern-based TCM treatment may be the variability in treatment principle. It is important that treatment principles should be included in future pattern-based TCM treatment studies. Further research on the link between TCM pattern, treatment principle, and treatment is warranted.

The effective rates of TCM treatments were quite similar across different TCM patterns. However, the effective rate summarized in this paper was based on case-series and clinical trials with inadequate study quality. Further studies with better methodological design should be performed to compare the efficacy of TCM treatment in different TCM patterns.

The present paper found that some Chinese herbs, such as Suanzaoren (Semen Z. spinosae), Fuling (Poria), Yejiaoteng (Caulis P. multiflori), Gancao (Radix Glycyrrhizae), and Baishao (Radix P. alba) were frequently used to treat insomnia regardless of the TCM pattern. In TCM theory, a standard Chinese herbal formula is consisted of “Sovereign,” “Minister,” “Assistant,” and “Courier” herbs. The Sovereign is used for treating the principal diseases; the Minister has synergistic effects with Sovereign herbs and helps to alleviate other accompanying symptoms; the Assistant is for enhancing the therapeutic effects and modulating the adverse effects of Sovereign and Minister herbs; while the Courier is used for harmonizing the actions of the others [23]. Suanzaoren (Semen Z. spinosae) and Yejiaoteng (Caulis P. multiflori) are classified as *Anshen* (mind tranquilizing) herbs and can act as Sovereign or Minister herbs in the treatment of insomnia. Fuling (Poria), Gancao (Radix Glycyrrhizae), and Baishao (Radix P. alba) have no specific tranquilizing effect in TCM and can be regarded as Assistant or Courier herbs in the herbal formulae commonly used for insomnia. The pharmacological basis for the different roles of the herbal agents in the treatment of insomnia remains to be determined. We also observed that a few herbs were specifically used for particular TCM patterns; for instance, Huangbo (Cortex Phellodendri), an *Qingre* (clear heat) agent, was exclusively used in *hyperactivity of fire due to yin deficiency*; Zhuru (Caulis Bambusae in Taeniam) and Dannanxing (Rhizome Arisaematis), which are classified as *Huatan* (resolve phlegm) herbs, were only used in *internal disturbance of phlegm heat*. As these herbs have no anxiolytic or sedative properties, we believe that the primary function of these herbs is not to directly treat insomnia but to alleviate the underlying causes of body imbalance.

For acupuncture-related therapies, we identified some acupoints, including Shenmen (HT7), Yintang (EX-HN3), Sanyinjiao (SP6), Baihui (GV20), Anmian (EX-HN22), and Sishencong (EX-HN1), which were generally used for insomnia regardless of the TCM pattern. All these commonly used acupoints are historically used for treating insomnia [24]. Similar to Chinese herbal medicine, we observed that a few acupoints were specifically used for particular TCM patterns; for example, Fenglong (ST40), based on the TCM theory, is used to remove phlegm and was exclusively used in *internal disturbance of phlegm heat*; Xingjian (LR2), which has the function of draining liver fire, was only used in *liver fire flaming upward*. We are unsure whether the few herbal agents and acupoints are essential for the treatment of insomnia in the particular TCM patterns. Further studies are needed to clarify the value of individual herbs and specific acupoints in pattern-based TCM treatments for insomnia. 

When traditional needle acupuncture treatment was combined with Chinese herbal medicine, Shenmen (HT7) was the most commonly used acupoint for the treatment of insomnia, but when traditional needle acupuncture was combined with auricular acupressure, Baihui (GV20) was more often used. Since there is an auricular point also called Shenmen which has a similar function to acupoint Shenmen (HT7) on the wrist, this may explain why Shenmen (HT7) was less frequently used when combined with auricular acupressure. Our finding suggested that the acupoint selection in traditional needle acupuncture might vary when it was combined with different TCM treatments.

The key shortcoming of incorporating TCM pattern diagnosis is that there is no standardization in terminology, and the diagnostic process is highly subjective. Considerable variability in TCM diagnosis has been found across practitioners in previous studies of menopausal syndrome, irritable bowel syndrome, rheumatoid arthritis, and low-back pain [25–28]. To further examine pattern-based TCM therapies, a standardized and reliable TCM pattern differentiation procedure should be explored. We have recently developed a standardized symptom checklist for TCM pattern differentiation in patients with insomnia [29]. The diagnostic tool, consisting of 13 sleep-related, 61 nonsleep-related, 11 tongue, and 7 pulse items, can be further validated for TCM diagnostic purpose. In addition, more than two-third of the reviewed studies did not report the criteria used for pattern diagnosis. This variability in the sets of rules for diagnosis is also a major reason for the disagreement between practitioners.

There are strengths as well as methodologic limitations of the study. Our data was generated from a systematic review of more than 200 articles involving almost 18000 subjects, which provided less biased results than those derived from TCM experts. The breadth of this paper would inevitably result in some uncertainties regarding the quality of data and accuracy of the TCM diagnosis. We included studies of different design, ranging from case series to RCTs; therefore, the comparison of efficacy of TCM treatments across TCM patterns should be treated with caution. In addition, most of the reviewed studies did not mention the TCM diagnostic procedure and diagnostic criteria used; hence, the quality of the diagnostic process was uncertain. Although a large number of studies were reviewed, information regarding the use of individual Chinese herbs and acupoints in some TCM patterns was still limited. 

Despite the limitations, the present study, for the first time, systematically and comprehensively summarized important data on pattern-based TCM treatments for insomnia. Since TCM pattern differentiation is an essential component of TCM treatment, our data should be useful for both clinical practice and research of TCM. More high quality studies incorporating TCM pattern differentiation and treatment principle are needed to examine the efficacy of TCM treatments and the additional benefit of pattern differentiation.

## Figures and Tables

**Table 1:** The 10 most common TCM patterns diagnosed in patients with insomnia.

TCM pattern	Chinese name	Number of subjects diagnosed with the TCM pattern (%) (total *N* = 17916)	Number of studies that examined the TCM pattern (%) (total *N* = 227)
*Deficiency of both the heart and spleen *	*心脾* *兩虛*	4056 (22.6)	180 (79.3)
*Hyperactivity of fire due to yin deficiency *	*陰虛* *火旺*	2926 (16.3)	132 (58.1)
*Liver-qi stagnation transforming into fire *	*肝鬱* *化火*	1817 (10.1)	91 (40.0)
*Heart-kidney noninteraction *	*心腎* *不交*	1157 (6.5)	60 (26.4)
*Internal disturbance of phlegm heat *	*痰熱* *內擾*	904 (5.0)	78 (34.4)
*Qi deficiency of the heart and gallbladder *	*心膽* *氣虛*	838 (4.7)	63 (27.8)
*Liver fire flaming upward *	*肝火* *上擾*	456 (2.5)	25 (11.0)
*Heart deficiency with timidity *	*心虛* *膽怯*	356 (2.0)	31 (13.7)
*Disharmony between spleen and stomach *	*脾胃* *不和*	325 (1.8)	19 (8.4)
*Stomach disharmony *	*胃腑* *不和*	162 (0.9)	17 (7.5)

**Table 2:** The commonly used Chinese herbal medicine for insomnia in subjects diagnosed with different TCM patterns^a^.

	*Deficiency of both the heart and spleen* (*心脾兩虛*)	*Hyperactivity of fire due to yin deficiency* (*陰虛火旺*)	*Liver-qi stagnation transforming into fire* (*肝鬱化火*)	*Heart-kidney noninteraction* (*心腎不交*)	*Internal disturbance of phlegm heat* (*痰熱內擾*)	*Qi deficiency of the heart and gallbladder* (*心膽氣虛*)	*Heart deficiency with timidity* (*心虛膽怯*)
Number of studies that examined the TCM pattern	*N* = 64	*N* = 53	*N* = 43	*N* = 13	*N* = 34	*N* = 20	*N* = 15
Commonly used Chinese herbal formula (*N*, % of studies that examined the TCM pattern)	Modified Gui Pi Tang (19, 29.7%)	Huang Lian E Jiao Tang (5, 9.4%)	Modified Long Dan Xie Gan Tang (6, 14.0%); Dan Zhi Xiao Yao San (6, 14.0%)	No formula was most frequently used	Modified Wen Dan Tang (10, 29.4%)	Modified An Shen Ding Zhi Wan (7, 35.0%)	No formula was most frequently used
Number of studies that provided TCM treatment principle	*N* = 11	*N* = 9	*N* = 8	*N* = 1	*N* = 6	*N* = 5	*N* = 1
Most commonly used treatment principle (*N*, % of studies that provided TCM treatment principle)	Nourish* heart *and* spleen* (5, 45.5%)	Nourish* yin *and suppress* fire* (5, 55.6%)	Soothe* liver *and purge* fire* (4, 50.0%)	Nourish* yin*, clear* fire,* and *coordinate heart* and *kidney* (1, 100.0%)	Clear* heat* and resolve* phlegm* (5, 83.3%)	Tonify* qi *and settle* fright* (5, 100.0%)	Tonify* qi *and settle* fright* (1, 100.0%)
Number of studies that provided the composition of herbal formula	*N* = 54	*N* = 43	*N* = 39	*N* = 11	*N* = 29	*N* = 16	*N* = 13
Composition of herbal formula (% of studies that provided the formula's composition)^b^							
Suanzaoren (Semen Z. spinosae)	**75.9%**	**58.1%**	48.7%	45.5%	**58.6%**	**68.8%**	**69.2%**
Fuling (Poria)	40.7%	34.9%	33.3%	27.3%	**75.9%**	**62.5%**	23.1%
Yejiaoteng (Caulis P. multiflori)	33.3%	46.5%	41.0%	/	41.4%	37.5%	**61.5%**
Gancao (Radix Glycyrrhizae)	27.8%	39.5%	**51.3%**	/	44.8%	37.5%	30.8%
Baishao (Radix P. alba)	24.1%	39.5%	46.2%	27.3%	20.7%	/	23.1%
Fushen (Poria cum Radix Pini)	33.3%	25.6%	23.1%	/	/	**50.0%**	38.5%
Hehuanpi (Cortex Albizziae)	22.2%	34.9%	30.8%	/	27.6%	/	46.2%
Yuanzhi (Radix Polygalae)	**53.7%**	23.3%	/	/	41.4%	**62.5%**	38.5%
Wuweizi (Fructus S. Chinensis)	24.1%	27.9%	/	27.3%	/	/	38.5%
Baiziren (Semen Platycladi)	20.4%	/	/	27.3%	/	/	23.1%
Shengdihuang (Radix Rehmanniae)	/	48.8%	30.8%	27.3%	/	/	/
Danggui (Radix A. sinensis)	**63.0%**	/	**51.3%**	/	/	/	/
Dangshen (Radix Codonopsis)	**51.9%**	/	/	/	/	43.8%	/
Baizhu (Rhizoma A. acrocephalae)	38.9%	/	20.5%	/	/	/	/
Honey-toasted Gancao (Radix Glycyrrhizae)	29.6%	/	/	/	/	25.0%	/
Dazao (Fructus Jujibae)	24.1%	/	/	27.3%	/	/	/
Huanglian (Rhizoma Coptidis)	/	41.9%	/	/	**51.7%**	/	/
Zhimu (Rhizoma Anemarrhenae)	/	30.2%	/	/	/	31.3%	/
Chaihu (Radix Bupleuri)	/	/	**64.1%**	/	/	/	23.1%
Zhizi (Fructus Gardeniae)	/	/	**53.8%**	/	27.6%	/	/
Zexie (Rhizoma Alismatis)	/	/	23.1%	27.3%	/	/	/
Zhenzhumu (Concha Margaritifera)	/	/	23.1%	/	/	/	46.2%
Shichangpu (Rhizoma A. Tatarinowii)	/	/	/	/	/	**56.3%**	46.2%
Longchi (Dens Draconis)	/	/	/	/	/	**50.0%**	30.8%
Huangqi (Radix Astragali)	**50.0%**	/	/	/	/	/	/
Ginger (Rhizoma Zingiberis)	20.4%	/	/	/	/	/	/
Longyanrou (Arillus Longan)	31.5%	/	/	/	/	/	/
Muxiang (Radix Aucklandiae)	31.5%	/	/	/	/	/	/
Huangbo (Cortex Phellodendri)	/	20.9%	/	/	/	/	/
Ejiao (Colla C asini)	/	41.9%	/	/	/	/	/
Longdancao (Radix Gentianae)	/	/	35.9%	/	/	/	/
Yujin (Radix Curcumae)	/	/	33.3%	/	/	/	/
Danpi (Cortex Moutan)	/	/	23.1%	/	/	/	/
Huangqin (Radix Scutellariae)	/	/	28.2%	/	/	/	/
Shudihuang (Radix Rehmanniae Praeparata)	/	/	/	27.3%	/	/	/
Dannanxing (Rhizome Arisaematis)	/	/	/	/	27.6%	/	/
Chenpi (Pericarpium C. Reticulatae)	/	/	/	/	44.8%	/	/
Zhuru (Caulis Bambusae in Taeniam)	/	/	/	/	**58.6%**	/	/
Zhishi (Fructus A. immaturus)	/	/	/	/	41.4%	/	/
Lime-water processed banxia (Rhizoma Pinelliae Preparatum)	/	/	/	/	44.8%	/	/
Renshen (Radix Ginseng)	/	/	/	/	/	25.0%	/
Hupo (Succinum)	/	/	/	/	/	37.5%	/
Chuanxiong (Rhizoma Chuanxiong)	/	/	/	/	/	25.0%	/
Longgu (Fossilia Ossis Mastodi)	/	/	/	/	/	/	23.1%

^a^Only those TCM patterns with more than 5 studies are presented.

^b^Individual Chinese herbs used in at least 50% of the studies on a particular TCM pattern are bolded.

**Table 3:** The mean effective rate of pattern-based TCM treatments for insomnia.

TCM pattern	Chinese name	Randomized controlled trials		Controlled trials		Case series	
		No. of studies	Mean effective rate in % (range, SD, 95% CI)	No. of studies	Mean effective rate in % (range, SD, 95% CI)	No. of studies	Mean effective rate in % (range, SD, 95% CI)
Chinese herbal medicine							
*Deficiency of both the heart and spleen *	*心脾* *兩虛*	9	88.9 (68.8–100.0, 11.4, 80.2–97.7)	3	97.6 (92.9–100.0, 4.1, 87.4–107.9)	17	86.5 (60.0–100.0, 12.1, 80.3–92.7)
*Hyperactivity of fire due to yin deficiency *	*陰虛* *火旺*	10	86.1 (66.7–100.0, 9.8, 79.1–93.1)	3	79.2 (50.0–94.7, 25.3, 16.3–142.1)	10	88.3 (66.7–100.0, 10.6, 80.7–95.9)
*Liver-qi stagnation transforming into fire *	*肝鬱* *化火*	6	95.0 (89.5–100.0, 4.7, 90.1–100.0)	2	96.7 (93.3–100.0, 4.7, 54.3–139.0)	8	79.1 (20.0–100.0, 28.6, 55.1–103.0)
*Heart-kidney noninteraction *	*心腎* *不交*	1	90.9 /	0	/	4	86.9 (84.9–89.7, 2.1, 83.5–90.3)
*Internal disturbance of phlegm heat *	*痰熱* *內擾*	5	84.4 (50.0–100.0, 20.7, 58.7–110.2)	1	100.0 /	8	86.3 (43.3–100.0, 18.0, 71.2–101.3)
*Qi deficiency of the heart and gallbladder *	*心膽* *氣虛*	0	/	1	66.7 /	5	75.3 (26.7–100.0, 30.0, 38.1–112.5)
*Liver fire flaming upward *	*肝火* *上擾*	0	/	0	/	1	80.0 /
*Heart deficiency with timidity *	*心虛* *膽怯*	5	79.4 (42.9–100.0, 22.0, 52.1–106.8)	1	87.5 /	1	77.8 /
*Disharmony between spleen and stomach *	*脾胃* *不和*	0	/	0	/	1	88.9 /
*Stomach disharmony *	*胃腑* *不和*	1	85.7 /	0	/	0	/
Acupuncture							
*Deficiency of both the heart and spleen *	*心脾* *兩虛*	4	94.4 (91.5–100.0, 3.9, 88.3–100.5)	3	83.4 (63.6–93.5, 17.1, 40.9–126.0)	13	90.8 (69.2–100.0, 8.7, 85.5–96.0)
*Hyperactivity of fire due to yin deficiency *	*陰虛* *火旺*	4	81.9 (50.0–96.7, 21.8, 47.2–116.5)	4	87.7 (73.3–98.0, 10.4, 71.2–104.2)	9	88.8 (41.7–100.0, 18.3, 74.7–102.9)
*Liver-qi stagnation transforming into fire *	*肝鬱* *化火*	1	75.0 /	2	97.6 (96.6–98.3, 1.0, 88.3–106.9)	6	81.5 (50.0–100.0, 19.8, 60.7–102.2)
*Heart-kidney noninteraction *	*心腎* *不交*	1	90.9 /	1	95.0 /	5	96.3 (87.5–100.0, 5.6, 89.4–103.2)
*Internal disturbance of phlegm heat *	*痰熱* *內擾*	2	94.5 (88.9–100.0, 7.8, 23.9–165.0)	1	95.0 /	5	95.0 (90.0–100.0, 4.7, 89.2–100.9)
*Qi deficiency of the heart and gallbladder *	*心膽* *氣虛*	0	/	3	76.9 (75.0–80.8, 3.3, 68.6–85.2)	4	75.0 (0.0–100.0, 50.0, −4.6–154.6)
*Liver fire flaming upward *	*肝火* *上擾*	2	92.9^a^ /	0	/	3	87.9 (63.0–100.0, 21.0, 35.7–140.1)
*Heart deficiency with timidity *	*心虛* *膽怯*	1	100.0 /	0	/	4	90.1 (70.0–100.0, 14.1, 67.6–112.6)
*Disharmony between spleen and stomach *	*脾胃* *不和*	0	/	0	/	3	98.1 (94.4–100.0, 3.2, 90.1–106.2)
*Stomach disharmony *	*胃腑* *不和*	2	100.0^a^ /	0	/	2	90.0 (80.0–100.0, 14.1, −37.1–217.1)

^a^Effective rate was the same in the 2 studies.

**Table 4:** The commonly used acupoints for insomnia in subjects diagnosed with different TCM patterns^a^.

	*Deficiency of both the heart and spleen* (*心脾兩虛*)	*Hyperactivity of fire due to yin deficiency* (*陰虛火旺*)	*Liver-qi stagnation transforming into fire* (*肝鬱化火*)	*Heart-kidney noninteraction* (*心腎不交*)	*Internal disturbance of phlegm heat* (*痰熱內擾*)	*Qi deficiency of the heart and gallbladder* (*心膽氣虛*)	*Liver fire flaming upward* (*肝火上擾*)	*Heart deficiency with timidity* (*心虛膽怯*)	*Disharmony between spleen and stomach* (*脾胃不和*)
No. of studies	*N* = 47	*N* = 33	*N* = 15	*N* = 21	*N* = 14	*N* = 17	*N* = 9	*N* = 6	*N* = 10
Acupoints^b^									
Shenmen (HT7)	**55.3%**	**54.5%**	40.0%	47.6%	**64.3%**	47.1%	**88.9%**	**50.0%**	40.0%
Yintang (EX-HN3)	29.8%	24.2%	20.0%	23.8%	35.7%	23.5%	33.3%	33.3%	30.0%
Sanyinjiao (SP6)	**53.2%**	42.4%	46.7%	42.9%	42.9%	29.4%	44.4%	33.3%	**50.0%**
Baihui (GV20)	34.0%	33.3%	20.0%	/	35.7%	35.3%	44.4%	33.3%	/
Anmian (EX-HN22)	25.5%	24.2%	/	23.8%	/	23.5%	**55.6%**	33.3%	20.0%
Sishencong (EX-HN1)	23.4%	24.2%	/	28.6%	21.4%	/	33.3%	33.3%	30.0%
Xinshu (BL15)	**72.3%**	39.4%	/	42.9%	42.9%	35.3%	/	**83.3%**	/
Neiguan (PC6)	34.0%	30.3%	33.3%	/	**57.1%**	/	/	33.3%	20.0%
Zhongwan (CV12)	21.3%	27.3%	33.3%	/	**57.1%**	23.5%	/	/	20.0%
Zusanli (ST36)	44.7%	/	/	/	**50.0%**	23.5%	22.2%	33.3%	30.0%
Fengchi (GB20)	23.4%	24.2%	26.7%	/	**50.0%**	/	44.4%	33.3%	/
Qihai (CV6)	/	24.2%	33.3%	/	42.9%	23.5%	/	/	20.0%
Shenshu (BL23)	/	45.5%	20.0%	38.1%	21.4%	/	/	/	/
Guanyuan (CV4)	/	21.2%	26.7%	/	42.9%	/	/	/	/
Ganshu (BL18)	/	/	40.0%	/	21.4%	/	33.3%	/	/
Pishu (BL20)	**51.1%**	/	/	/	28.6%	/	/	/	/
Danshu (BL19)	/	/	/	/	/	35.3%	/	**83.3%**	/
Weishu (BL21)	/	/	/	/	21.4%	/	/	/	30.0%
Taixi (Kl13)	/	42.4%	/	42.9%	/	/	/	/	
Zhaohai (KI6)	/	21.2%	/	/	/	/	11.1%	/	/
Xiawan (CV10)	/	/	20.0%	/	21.4%	/	/	/	/
Shenmai (BL62)	/	/	/	/	21.4%	/	11.1%	/	/
Cuanzhu (BL2)	/	/	/	/	21.4%	/	/	33.3%	/
Taichong (LR3)	/	/	40.0%	/	/	/	**100.0%**	/	/
Fenglong (ST40)	/	/	/	/	**57.1%**	/	/	/	/
Qingming (BL1)	/	/	/	/	21.4%	/	/	/	/
Xingjian (LR2)	/	/	/	/	/	/	**66.7%**	/	/
Hegu (Ll4)	/	/	/	/	/	/	22.2%	/	/
Xuehai (SP10)	/	/	/	/	/	/	22.2%	/	/
Yanglingquan (GB34)	/	/	/	/	/	/	/	33.3%	/
Yuyao (EX-HN4)	/	/	/	/	/	/	/	33.3%	/
Sibai (ST2)	/	/	/	/	/	/	/	33.3%	/
Daling (PC7)	/	/	/	/	/	/	/	33.3%	/
Jianjing (GB21)	/	/	/	/	/	/	/	33.3%	/
Tianshu (ST25)	/	/	/	/	/	/	/	/	20.0%

^a^Studies on *stomach disharmony* are too few to give reliable information.

^b^Acupoints that were used in at least 50% of the studies on a particular TCM pattern are bolded.

**Table 5:** The commonly used Chinese herbal medicine and acupoints in the combined Chinese herbal medicine and traditional needle acupuncture treatment for insomnia in subjects diagnosed with different TCM patterns^a^.

	*Deficiency of both the heart and spleen* (*心脾兩虛*)	*Hyperactivity of fire due to yin deficiency* (*陰虛火旺*)	*Liver-qi stagnation transforming into fire* (*肝鬱化火*)
No. of studies that examined the TCM pattern	*N* = 12	*N* = 6	*N* = 7
No. of studies that provided TCM treatment principle	*N* = 5	*N* = 4	*N* = 3
Most frequently used TCM treatment principle (N, % of studies that provided TCM treatment principle)	Nourish *heart* and *spleen* (3, 60.0%)	Nourish *yin* and suppress *fire* (4, 100.0%)	Soothe *liver* and purge* fire* (2, 66.7%)
Individual Chinese herbs^b^			
Danggui (Radix A. sinensis)	**75.0%**	33.3%	**71.4%**
Chaihu (Radix Bupleuri)	25.0%	33.3%	**85.7%**
Gancao (Radix Glycyrrhizae)	41.7%	33.3%	42.9%
Suanzaoren (Semen Z. spinosae)	**100.0%**	**83.3%**	/
Huangqin (Radix Scutellariae)	*/ *	**66.7%**	**85.7%**
Baishao (Radix P. alba)	*/ *	**66.7%**	28.6%
Fushen (Poria cum Radix Pini)	33.3%	/	28.6%
Shengdihuang (Radix Rehmanniae)	*/ *	33.3%	**57.1%**
Yejiaoteng (Caulis P. multiflori)	41.7%	**50.0% **	/
Fuling (Poria)	**50.0%**	33.3%	/
Hehuanhua (Flos Albiziae)	**50.0%**	33.3%	/
Honey-toasted Gancao (Radix Glycyrrhizae)	33.3%	33.3%	/
Baizhu (Rhizoma A. macrocephalae)	**83.3%**	/	/
Huangqi (Radix Astragali)	**75.0%**	/	/
Yuanzhi (Radix Polygalae)	**66.7%**	/	/
Muxiang (Radix Aucklandiae)	**58.3%**	/	/
Longyanrou (Arillus Longan)	**50.0%**	/	/
Dazao (Fructus Jujibae)	41.7%	/	/
Renshen (Radix Ginseng)	33.3%	/	/
Wuweizi (Fructus S. Chinensis)	33.3%	/	/
Baiziren (Semen Platycladi)	25.0%	/	/
Chuanxiong (Rhizoma Chuanxiong )	25.0%	/	/
Dangshen (Radix Codonopsis)	25.0%	/	/
Ginger (Rhizoma Z. recens)	25.0%	/	/
Huanglian (Rhizoma Coptidis)	/	**83.3%**	/
Egg yolk (Vitellus Galli)	/	**50.0%**	/
Ejiao (Colla C. asini)	/	**50.0%**	/
Zhizi (Fructus Gardeniae)	/	/	**100.0%**
Cheqianzi (Semen Plantaginis)	/	/	**71.4%**
Longgu (Fossilia Ossis Mastodi)	/	/	**71.4%**
Muli (Concha Ostreae)	/	/	**71.4%**
Longdancao (Radix Gentianae)	/	/	**57.1%**
Mutong (Caulis C. armandii)	/	/	**57.1%**
Zexie (Rhizoma Alismatis)	/	/	**57.1%**
Hehuanpi (Cortex Albizziae)	/	/	42.9%
Yujin (Radix Curcumae)	/	/	42.9%
Danpi (Cortex Moutan)	/	/	28.6%
Acupoints^b^			
Shenmen (HT7)	**100.0%**	**100.0%**	**100.0%**
Sanyinjiao (SP6)	**83.3%**	**50.0%**	**71.4%**
Neiguan (PC6)	**58.3%**	**50.0%**	**57.1%**
Sishencong (EX-HN1)	41.7%	33.3%	**71.4%**
Baihui (GV20)	33.3%	**50.0%**	42.9%
Zusanli (ST36)	**58.3%**	33.3%	28.6%
Taichong (LR3)	/	**66.7%**	**71.4%**
Hegu (Ll4)	/	33.3%	**57.1%**
Xinshu (BL15)	**75.0% **	/	/
Pishu (BL20)	**66.7%**	/	/
Taixi (Kl13)	/	**66.7%**	/
Daling (PC7)	/	**50.0%**	/
Shenshu (BL23)	/	33.3%	/
Taiyuan (LU9)	/	33.3%	/
Zhaohai (Kl6)	/	33.3%	/
Anmian (EX-HN22)	/	/	28.6%
Fengchi (GB20)	/	/	28.6%
Ganshu (BL18)	/	/	28.6%
Houxi (SI3)	/	/	28.6%
Shenmai (BL62)	/	/	28.6%

^a^Other TCM patterns are not listed because studies are too few to give reliable information.

^b^Individual Chinese herbs and acupoints used in at least 50% of the studies on a particular TCM pattern are bolded.

**Table 6:** The most commonly used acupoints and auricular points in the combined traditional needle acupuncture and auricular acupressure treatment for insomnia in subjects diagnosed with different TCM patterns^a^.

	*Deficiency of both the heart and spleen* (*心脾兩虛*)	*Hyperactivity of fire due to yin deficiency* (*陰虛火旺*)	*Liver-qi stagnation transforming into fire* (*肝鬱化火*)	*Heart-kidney noninteraction* (*心腎不交*)	*Qi deficiency of the heart and gallbladder* (*心膽氣虛*)
No. of studies that examined the TCM pattern	*N* = 15	*N* = 8	*N* = 6	*N* = 8	*N* = 6
Acupoints^b^					
Sanyinjiao (SP6)	**66.7% **	**50.0% **	**83.3% **	**62.5% **	**66.7% **
Baihui (GV20)	**66.7% **	**75.0% **	**83.3% **	**62.5% **	**50.0% **
Shenmen (HT7)	**66.7%**	**62.5%**	33.3%	**75.0% **	**66.7% **
Sishencong (EX-HN1)	**53.3% **	**62.5% **	**66.7% **	25.0%	**50.0% **
Neiguan (PC6)	46.7%	37.5%	**50.0% **	**50.0% **	33.3%
Anmian (EX-HN22)	33.3%	37.5%	/	37.5%	33.3%
Zusanli (ST36)	40.0%	25.0%	33.3%	/	/
Taixi (Kl13)	/	**75.0% **	/	**62.5% **	33.3%
Taichong (LR3)	/	25.0%	**83.3% **	/	/
Xinshu (BL15)	46.7%	/	/	**75.0% **	/
Fengchi (GB20)	20.0%	25.0%	/	/	/
Shenshu (BL23)	/	25.0%	/	**50.0% **	/
Pishu (BL20)	40.0%	/	/	/	/
Benshen (GB13)	/	25.0%	/	/	/
Shenting (GV24)	/	25.0%	/	/	/
Yintang (EX-HN3)	/	37.5%	/	/	/
Ganshu (BL18)	/	/	33.3%	/	/
Dazhui (GVl4)	/	/	/	25.0%	/
Shenmai (BL62)	/	/	/	25.0%	/
Zhaohai (Kl6)	/	/	/	25.0%	/
Danshu (BLl9)	/	/	/	/	33.3%
Auricular points^b^					
Shenmen	**100.0% **	**100.0% **	**100.0%**	**100.0% **	**100.0% **
Subcortex	**80.0% **	**100.0% **	**83.3% **	**50.0% **	**100.0% **
Heart	**73.3% **	**62.5% **	**50.0% **	**62.5% **	**83.3% **
Kidney	46.7%	**50.0% **	33.3%	**75.0% **	**66.7% **
Sympathesis	46.7%	**62.5% **	**66.7% **	25.0%	**50.0% **
Spleen	**66.7% **	25.0%	33.3%	37.5%	**50.0% **
Liver	26.7%	37.5%	**66.7% **	25.0%	**66.7% **
Endocrine	26.7%	**62.5% **	**50.0% **	/	**50.0% **
Brain	40.0%	25.0%	/	**50.0% **	33.3%
Occiput	20.0%	25.0%	33.3%	25.0%	/
Neurasthenia point	26.7%	37.5%	33.3%	/	/
Stomach	26.7%	/	/	/	/
Gallbladder	/	/	33.3%	/	/

^a^Other TCM patterns are not listed because studies are too few to give reliable information.

^b^Acupoints and auricular points used in at least 50% of the studies on a particular TCM pattern are bolded.
